# Feedback activation of NF-KB signaling leads to adaptive resistance to EZH2 inhibitors in prostate cancer cells

**DOI:** 10.1186/s12935-021-01897-w

**Published:** 2021-04-01

**Authors:** Mengyuan Jin, Jiachen Duan, Wei Liu, Jing Ji, Bin Liu, Mingzhi Zhang

**Affiliations:** 1 Department of Oncology, The First Affiliated Hospital of Zhengzhou University, Zhengzhou, 450052 China; 2 Department of Urology, The First Affiliated Hospital of Zhengzhou University, Zhengzhou, 450052 China; 3 Academy of Medical Sciences, Zhengzhou University, Zhengzhou, 450052 China; 4 Jiangsu Key Laboratory of Marine Pharmaceutical Compound Screening, College of Pharmacy, Jiangsu Ocean University, Lianyungang, 222005 China; 5 Lymphoma Diagnosis and Treatment Center of Henan Province, Zhengzhou, 450000 China

**Keywords:** EZH2, NF-κB, Prostate cancer (PCa), SOX9, TNFRSF11A (RANK)

## Abstract

**Background:**

Prostate cancer (PCa) is the most common malignant tumor in developed countries, which has seriously threatened men’s lifestyle and quality of life. The up-regulation of EZH2 is associated with advanced PCa and poor prognosis, making it a promising therapeutic target. However, the EZH2 inhibitors-based treatment is basically ineffective against PCa, which limits its clinical application.

**Methods:**

Microarray data (GSE107779) from LNCaP cells treated with either siRNA against EZH2 or a EZH2 inhibitor EPZ6438 was analyzed by Limma R package. Western blot, real-time PCR and luciferase reporter assays were used to determine the EZH2-SOX9-TNFRSF11A axis and the activity of NF-κB signaling in PCa cells. CCK-8 assay was used to determine the viability of PCa cells following various treatments.

**Results:**

Genetic ablation or pharmacological inhibition of EZH2 leads to feedback activation of NF-κB signaling in PCa cells. EZH2-dependent SOX9 expression regulates the activation of NF-κB signaling. TNFRSF11A, also known as receptor activator of NF-κB (RANK), is a downstream target of SOX9 in PCa cells. SOX9 recognizes two putative SOX9 response elements in the promoter region of TNFRSF11A gene to drive TNFRSF11A expression and downstream NF-κB signaling activation. Suppression of the NF-κB signaling by either TNFRSF11A silencing or BAY11-7082 treatment rendered PCa cells to EZH2 inhibitors.

**Conclusion:**

Collectively, our finding reveals a EZH2-SOX9-TNFRSF11A axis in the regulation of activity of NF-κB signaling in PCa cells and suggests that a combination of EZH2 inhibitors and BAY11-7082 would be an effective approach for the treatment of PCa patients in the future.

**Supplementary Information:**

The online version contains supplementary material available at 10.1186/s12935-021-01897-w.

## Background

Prostate cancer (PCa) is the most common malignant tumor in men in developed countries such as Europe and the United States [1]. The incidence of PCa in the United States has now exceeded that of lung cancer [1]. Compared with developed countries, the incidence of PCa in China is relatively low, ranking the sixth among male malignancies [2]. However, with the rapid economic growth of China, the incidence of PCa has also increased rapidly in recent years [3]. In developing country, due to the insidious symptoms, many patients have been diagnosed in an advanced stage and the therapeutic effect of advanced PCa is usually very poor. Thus, it is crucial to develop efficient treatments for advanced PCa patients.

EZH2, the enzymatic subunit of the poly-comb repressive complex 2 (PRC2), plays a critical role in catalyzing the methylation of the lysine 27 of histone H3 (H3K27) [4]. Aberrant activation or expression of EZH2 are believed to drive a H3K27 methylation (H3K27me)-dependent cell growth, making EZH2 a promising target for cancer therapy [5]. Two EZH2 inhibitors, EPZ-6438 and GSK126, showed preliminary benefits in some hematological malignances with constitutive enzymatic activity of EZH2 [6, 7]. GSK126 could decrease global H3K27me3 levels and reactivate silenced PRC2 target genes. It could also effectively inhibit the proliferation of EZH2 mutant DLBCL cell lines and inhibits xenografts growth in mice [7]. Treatment of EZH2-mutant NHL xenografts in mice with EPZ-6438 also causes tumor growth inhibition [8]. Although the up-regulation of EZH2 is associated with advanced PCa and poor prognosis [9], the EZH2 inhibitors-based treatment is basically ineffective for PCa [10], which limits its clinical application.

NF-κB is a major regulator of crucial cell processes, such as inflammation, proliferation, and apoptosis [11]. The NF-κB signaling can be triggered by several stimuli, including inflammatory cytokines such as tumor necrosis factor-α (TNF-α) and interleukin-1β (IL-1β), which initiate the classical pathway [12]. Interestingly, TNF-α and IL-1β, are not only NF-κB activators, but also NF-κB targets that forming a positive feedback loop to promote its continuous activation [12]. Abnormally activated NF-κB signaling could cause PCa to evade treatment with pro-apoptotic drugs [13].

In the present study, we found that inhibition of EZH2 inhibition led to feedback activation of NF-κB signaling in PCa cells and uncovered a molecular mechanism of adaptive resistance to EZH2 inhibitors in PCa. Our results suggest a combinatorial approach with both EZH2 and NF-κB inhibitors for treating PCa.

## Material and methods

### Data acquisition

The microarray data (GSE107779 and GSE76441) were downloaded from the Gene Expression Omnibus (GEO) database and differentially expressed genes were screened via the R software Linear Models for Microarray and RNA-Seq Data (Limma) package (http://bioconductor.org/packages/Limma/). GSE107779 contains gene expression profile microarrays from LNCaP cells treated with either siRNA against EZH2 or a EZH2 inhibitor EPZ6438. GSE76441 contains gene expression profile microarrays from VCAP cells transfected with con-siRNA or siRNA against SOX9. We chose a p < 0.05 and |log2FC| ≥ 1 as the cutoff criteria.

### Cell culture, tissue samples and reagents

HEK293T and PCa cell lines LNCaP, PC3, 22RV1 and Du-145 cells were purchased from Cell Bank of Shanghai Institute of Biological Science (SIBS, CAS, Shanghai, China). Cells were cultured in Dulbecco’s modified Eagle’s medium (DMEM) or Gibco Roswell Park Memorial Institute (RPMI) 1640, supplemented with 10% FBS, 100 units/mL penicillin, and 100 mg/mL streptomycin. EPZ-6438, GSK126 and BAY11-7082 were purchased from Selleck. DMSO was purchased from Beyotime Institute of Biotechnology.

### RNA isolation, real-time PCR and RNA interference

The Trizol (Invitrogen, USA) was used to extract the total RNA from PCa cells. The mRNA is then transcribed into cDNA by using the reverse transcription kit (Promega, USA). Real-time PCR was performed on Light Cycler 480 (Roche, Switzerland). The 2 − ΔΔCt method was used for relative quantification using GAPDH as the endogenous control. Primers were listed as follows: IL1B forward, 5′-ATGATGGCTTATTACAGTGGCAA-3′; reverse, 5′-GTCGGAGATTCGTAGCTGGA′; TNFA forward, 5′-TCAGATCATCTTCTCGAACCCC-3′; reverse, 5′-ATCTCTCAGCTCCACGCCAT; SOX9 forward, 5′-AGCGAACGCACATCAAGAC-3′; reverse, 5′-CTGTAGGCGATCTGTTGGGG; TNFRSF11A forward, 5′-AGATCGCTCCTCCATGTACCA-3′; reverse, 5′-GCCTTGCCTGTATCACAAACTTT; EZH2 forward, 5′-AATCAGAGTACATGCGACTGAGA-3′; reverse, 5′-GCTGTATCCTTCGCTGTTTCC; siRNAs were ordered as RPHPLC-purified duplexes from GenePharma. The sequences of the siRNAs used in this study are shown as follows: EZH2-siRNA-1 (GCUGAAGCCUCAAUGUUUA); EZH2-siRNA-2 (GAAUGGAAACAGCGAAGGA), SOX9-siRNA (5′-UGGGCAAGCUCUGGAGACUUCUGAA), TNFRSF11A-siRNA (CACCAAAUGAACCCCAUGUUUAC) and scrambled control siRNA (GGUAGCGCCAAUCCUUACGUCUCUU).

### Western blotting and antibodies

Cells with virous treatments were lysed with 2 × SDS loading buffer. The lysate was heated at 95 °C for 10 min and precipitate was removed by centrifugation at high speed. The supernatant was separated by 10% SDS–PAGE and transferred to nitrocellulose (NC) membranes. The membranes were blocked with 5% non-fat milk for 1 h, and incubated with indicated primary antibodies overnight at 4 °C. The following primary antibodies were used: anti-SOX9 (sc-166505, Santa Cruz, USA), anti-TNFRSF11A (RANK) (sc-374360, Santa Cruz, USA) and anti-GAPDH (sc-47724, Santa Cruz, USA). The membranes were then washed with PBS for three times followed by incubation with secondary antibodies for 45 min. The signals were visualized by an ImageQuant LAS 4000 system (GE Healthcare).

### Cell viability assay

Cells were seeded in 96-well plates overnight and treated with indicated compounds for 36–48 h. CCK8 assay (Beyotime Institute of Biotechnology, China) was then carried out. The absorbance (optical density, OD) was read at a wavelength of 450 nm on an ELISA plate reader.

### Plasmids, luciferase and ChIP assay

SOX9 were amplified from PC3 cells by PCR and cloned into the pBabe-3 × Flag retroviral vector. The Lipofectamine 2000 Transfection Reagent was used for gene delivery according to the manufacturer’s instructions. A luciferase expression vector controlled by a promoter containing three repeats of the NF-κB response element was obtained from Beyotime Institute of Biotechnology. The pSV40-Renilla plasmid was purchased from Promega. The promoter region of TNFRSF11A gene was amplified from the genomic DNA of PC3 cells and inserted into pGL4.15 vector (Promega, Madison, WI, USA). For the activity of NF-κB, cells were plated in triplicates in a 24-well plate and then transiently transfected with pNFκB-luc and the internal control plasmid pSV40-Renilla for 12 h. Cells were then treated with indicated drugs. For the transcriptional regulation of TNFRSF11A by SOX9, cells were plated in triplicates in a 24-well plate and then transiently transfected with SOX9/pSV40-Renilla plasmids together with either WT or mutant TNFRSF11A-luc plasmids for 36 h. The firefly luciferase reporter activity was assayed 36–48 h after transfection, using the Dual Luciferase Assay System (Promega, Madison, WI, USA) according to the manufacturer’s instructions. The firefly luciferase luminescence data were normalized by the Renilla luciferase luminescence data. For Chip assay, a ChIP assay kit (Upstate, Billerica, MA, USA) was used. Cells were fixed with formaldehyde and DNA was sheared to fragments at 100–500 bp by sonication. The immunocomplexes were then incubated and precipitated with antibodies against SOX9 or normal serum IgG overnight and rotationally incubation at 4 °C.

### Statistical analyses

All experiments are repeated at least three times. The data is represented by the mean ± standard deviation (SD). The statistics and graphing of the data were performed by GraphPad Prism 8.0 software. The difference between the two groups was assessed by Student's t test. Two-way ANOVA.

For more than two groups, the difference was assessed by two-way ANOVA followed by Tukey’s multiple comparisons test. P < 0.05 was considered statistically significant. *P < 0.05, **P < 0.01 and ***P < 0.001.

## Results

### In silico identification of EZH2-regulated genes in PCa cells

**Fig. 1:**
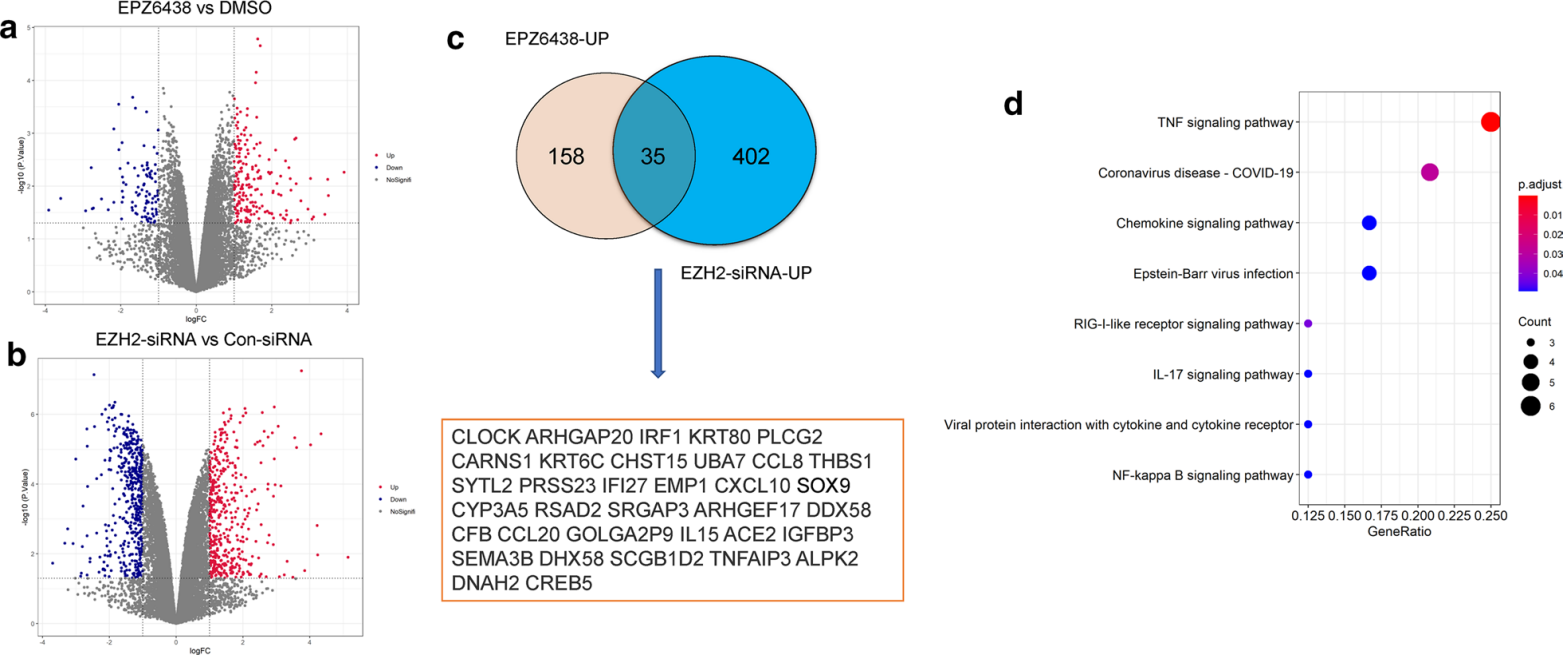
In silico identification of EZH2-regulated genes in PCa cells. **a** The microarray data from LNCaP cells treated with DMSO or EPZ6438 was used to examine the differentially expressed mRNAs. 887 differentially expressed genes including 437 up-regulated and 450 down-regulated were identified by the Limma R package and selected to draw a volcano plot. **b** The microarray data from LNCaP cells transfected with con-siRNA or EZH2-siRNA was used to examine the differentially expressed mRNAs. 289 differentially expressed genes including 193 up-regulated and 96 down-regulated were identified by the Limma R package and selected to draw a volcano plot. **c** The intersection of the common up-regulated genes in LNCaP cells after EZH2 inhibition or knockdown identified 35 potential substrates of EZH2. **d** The KEGG pathway analysis of these potential EZH2 substrates

To investigate the resistance mechanism of PCa cells to EZH2 inhibitors, we first analyzed the potential substrate genes of EZH2 in PCa by using a Gene Expression Omnibus (GEO) database (GSE107779) [14]. GSE107779 contains gene expression profile microarrays from LNCaP cells treated with either siRNA against EZH2 or a EZH2 inhibitor EPZ6438. We proposed that the common up-regulated genes in LNCaP cells after EZH2 inhibition or knockdown might represent the potential targets of EZH2. We chose a p < 0.05 and |log2FC| ≥ 1 as the cutoff criteria. We found that 437 genes were up-regulated and 450 genes were down-regulated after EZH2 was silenced (Fig. 1a; Additional file 1: Table S1), and 193 genes were up-regulated and 96 genes were down-regulated after EZH2 was inhibited (Fig. 1b; Additional file 2: Table S2). Using the above-mentioned criterion, we identified 35 potential substrates of EZH2 (Fig. 1c). This approach yielded many known EZH2 substrate genes, such as IRF1 [15], CXCL10 [16] and SOX9 [17], as well as several previously unknown putative substrate genes, such as CLOCK and TNFRSF11A (Fig. 1c). The KEGG analysis of these genes found that inflammatory pathways such as TNFA and NF-κB signaling pathways were significantly enriched (Fig. 1d), suggesting that EZH2 may inhibit inflammatory pathways in PCa cells (Additional file 2: Table S2).

### Genetic ablation or pharmacological inhibition of EZH2 leads to feedback activation of the NF-κB signaling in PCa cells

**Fig. 2:**
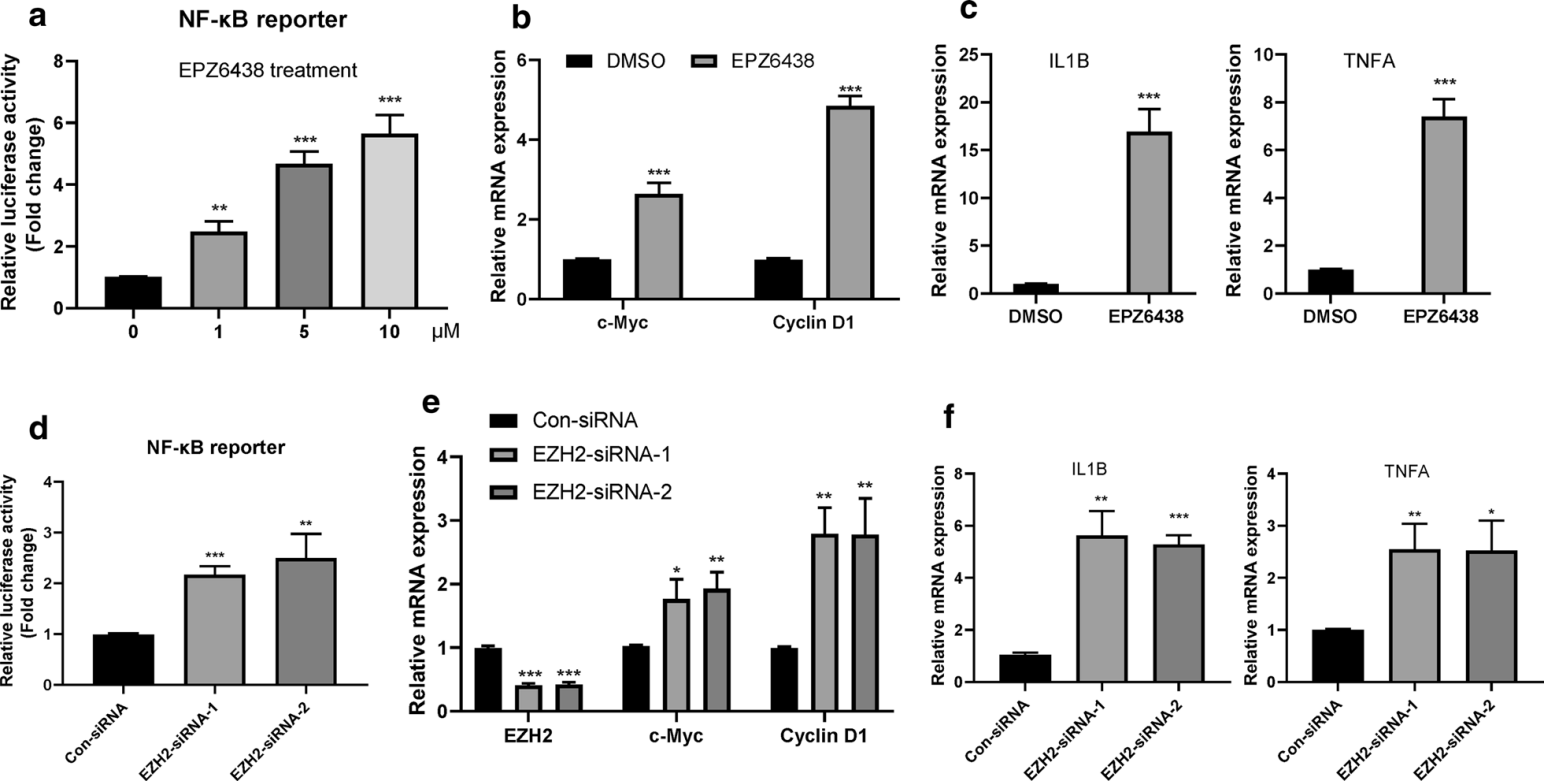
Genetic ablation or pharmacological inhibition of EZH2 leads to feedback activation of the NF-κB signaling in PCa cells.** a** LNCaP cells were transfected with pGL3-NFkB-Luc and pSV40-renilla plasmids for 12 h and then treated with indicated dose of EPZ6438 for additional 24 h. The luciferase activity was then measured. **P < 0.01, ***P < 0.001. **b** Relative mRNA expression levels of the two NF-κB downstream target genes c-Myc and Cyclin D1 in 10 μM EPZ6438 treated LNCaP cells were determined by real-time PCR assay. ***P < 0.001. **c** Relative mRNA expression levels of the two NF-κB downstream inflammatory cytokines IL1B and TNFA in 10 μM EPZ6438 treated LNCaP cells were determined by real-time PCR assay. ***P < 0.001. **d**. LNCaP cells were transfected with indicated siRNAs for 12 h, and then co-transfected with pGL3-NFkB-Luc and pSV40-renilla plasmids for 24 h. The luciferase activity was then measured. **P < 0.01, ***P < 0.001. **e** LNCaP cells were transfected with indicated siRNAs for 36 h, the relative mRNA expression levels of EZH2, c-Myc and Cyclin D1 were determined by real-time PCR assay. *P < 0.05, **P < 0.01, ***P < 0.001. **f** LNCaP cells were transfected with indicated siRNAs for 36 h, the relative mRNA expression levels of IL1B and TNFA were determined by real-time PCR assay. *P < 0.05, **P < 0.01, ***P < 0.001

Since the activation of NF-κB signaling is critical to the survival of tumor cells, we speculate that inhibition of EZH2 in PCa cells may cause feedback activation of NF-κB signaling. To this end, we first test the activity of a luciferase reporter regulated by the NF-κB-consensus motif in LNCaP cells with or without EPZ6438 treatment. We found that EPZ6438 treatment increased the activity of NF-κB in a dose-dependent manner (Fig. 2a). Moreover, EPZ6438 treatment further induced the expression of NF-κB downstream target genes (Fig. 2b) and inflammatory cytokines (Fig. 2c). The treatment of PC3 cells with another EZH2 inhibitor GSK126 also yielded similar phenomena (Additional file 5: Fig. S1A–C). Similarly, silencing EZH2 in LNCaP cells by two different siRNAs could also lead to the activation of NF-κB signaling, including the enhanced NF-kB luciferase activity (Fig. 2d), increased expression of NF-κB downstream target genes (Fig. 2e) and inflammatory cytokines (Fig. 2f). Therefore, these data indicate that inhibition or knockdown of EZH2 can result in feedback activation of NF-κB signaling in PCa cells.

### Suppression of the NF-κB signaling pathway reduces the proliferation and viability of PCa cells treated with EZH2 inhibitors

**Fig. 3:**
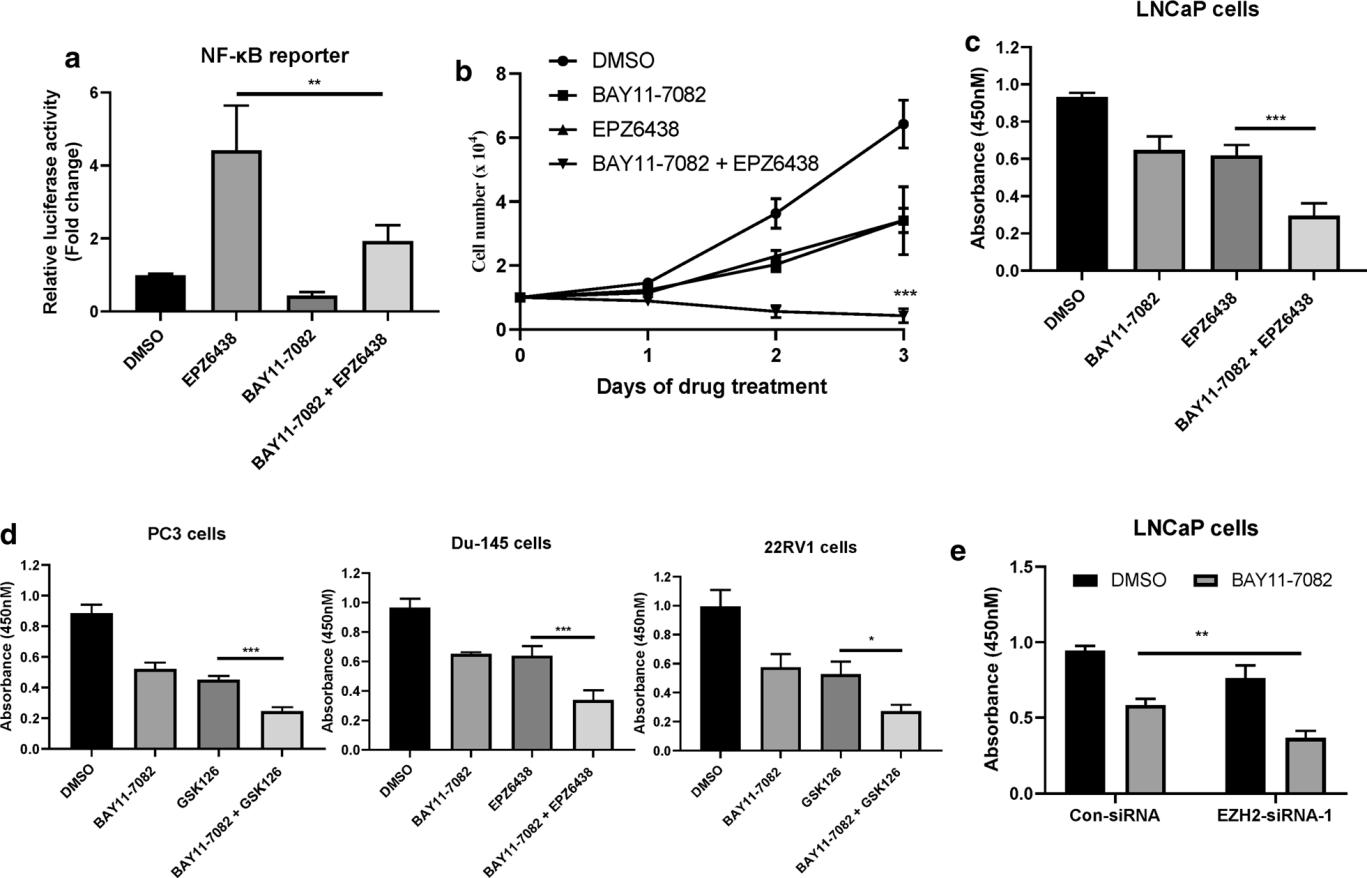
Suppression of the NF-κB signaling pathway reduces the proliferation and viability of PCa cells treated with EZH2 inhibitors. **a** LNCaP cells were transfected with pGL3-NFkB-Luc and pSV40-renilla plasmids for 12 h and then treated with either 10 μM EPZ6438 and/or 10 μM BAY11-7082 for additional 24 h. The luciferase activity was then measured. **P < 0.01. **b** The cell growth curve of LNCaP cells treated with or without either 5 μM EPZ6438 and/or 5 μM BAY11-7082 for the indicated days. ***P < 0.001. **c** LNCaP cells were treated with or without either 10 μM EPZ6438 and/or 10 μM BAY11-7082 for 24 h. CCK-8 was then added and the absorbance at A450 was determined by a microplate reader. *** P < 0.001. **d** PCa cell lines PC3, 22RV1 and Du-145 were treated with or without either 10 μM GSK126/EPZ6438 and/or 10 μM BAY11-7082 for 24 h. CCK-8 was then added and the absorbance at A450 was determined by a microplate reader. ***P < 0.001. **e** LNCaP cells were transfected with indicated siRNAs for 24 h and then treated with or without 10 μM BAY11-7082 for additional 24 h. The luciferase activity was then measured. ***P < 0.001

The inhibition of EZH2 leads to feedback activation of NF-κB signaling, which may promote the growth of PCa cells and lead to their resistance to EZH2 inhibitors. We found that BAY11-7082 treatment can significantly inhibit the activation of NF-κB signaling induced by EPZ6438 in LNCaP cells (Fig. 3a) [18]. Moreover, the combination of BAY11-7082 and EPZ6438 inhibited the proliferation and decreased the viability of LNCaP cells when compared to cells treated with EPZ6438 or BAY11-7082 alone (Fig. 3b, c). Similarly, BAY11-7082 administration also rendered other PCa cancer cell lines including PC3, Du-145 and22RV1 cells sensitive to EZH2 inhibitors treatment (Fig. 3d). Consistent with these results, silencing the expression of EZH2 also sensitized LNCaP cells to BAY11-7082 treatment (Fig. 3e). Together, these data suggested that feedback activation of NF-κB signaling leads to adaptive resistance to EZH2 inhibitors in PCa cells.

### EZH2-dependent SOX9 expression regulates the activation of NF-κB signaling

**Fig. 4:**
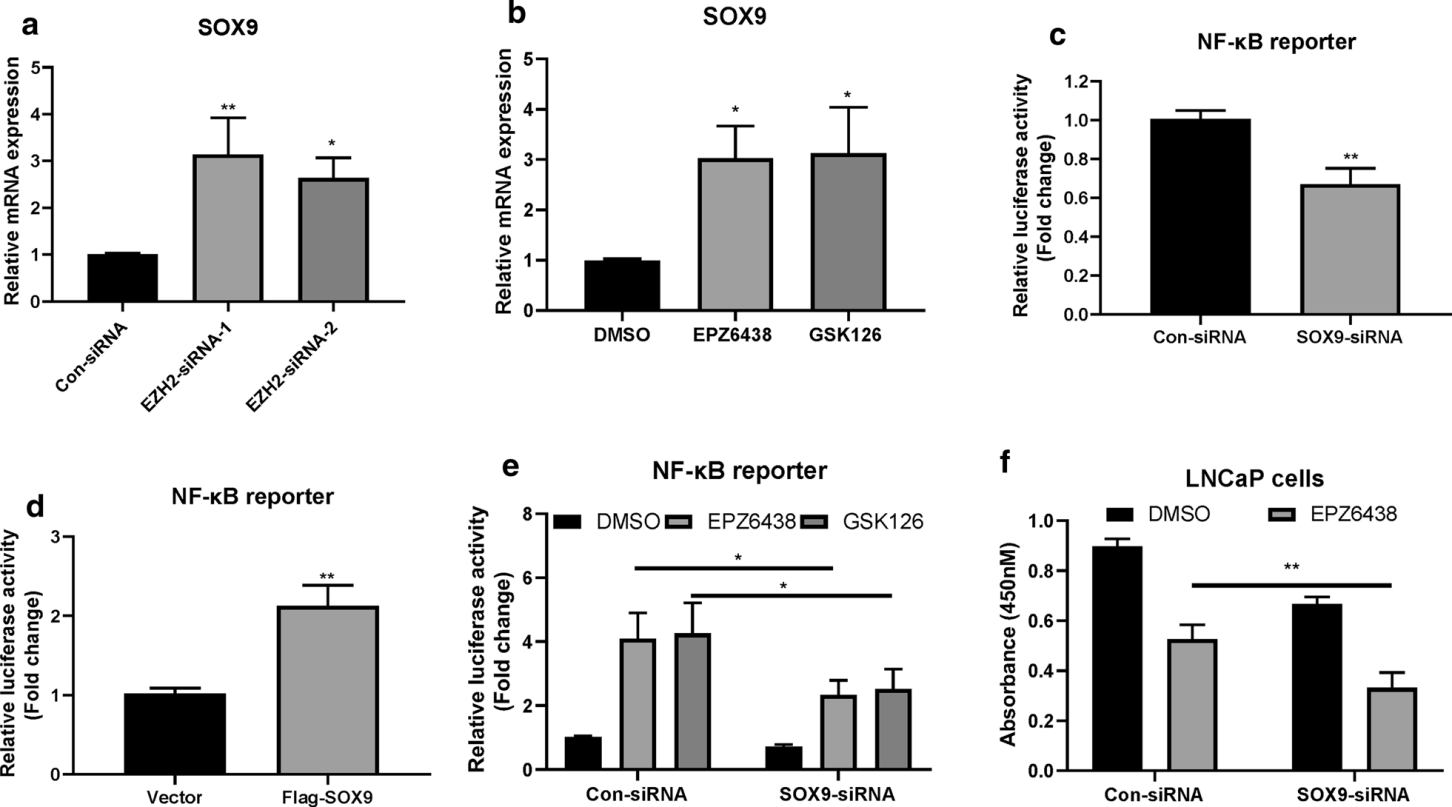
EZH2-dependent SOX9 expression regulates the activation of NF-κB signaling. **a** LNCaP cells were transfected with indicated siRNAs for 36 h, the relative mRNA expression levels of SOX9 were determined by real-time PCR assay. *P < 0.05, **P < 0.01. **b** LNCaP cells were treated with 10 μM EPZ6438 or GSK126 for 24 h. The relative mRNA levels of SOX9 were then determined. *P < 0.05. **c** LNCaP cells were transfected with indicated siRNAs for 12 h, then co-transfected with pGL3-NFkB-Luc and pSV40-renilla plasmids for 24 h. The luciferase activity was then measured. **P < 0.01. **d** LNCaP cells were co-transfected with indicated plasmids for 24 h. The luciferase activity was then measured. **P < 0.01. **e** LNCaP cells were transfected with indicated siRNAs for 24 h and then treated with or without 10 μM EPZ6438 or GSK126 for additional 24 h. The luciferase activity was then measured. **P < 0.05. **f** LNCaP cells were transfected with indicated siRNAs for 24 h and then treated with or without 10 μM EPZ6438 for additional 24 h. CCK-8 was then added and the absorbance at A450 was determined by a microplate reader. **P < 0.01

Next, we investigated how the inhibition of EZH2 leads to NF-κB signaling activation. It has been reported that EZH2-induced SOX9 downregulation participated in the cartilaginous endplate (CEP) degeneration process [17]. We also found that EZH2 could regulate the expression of SOX9 in PCa cells. Indeed, either knocking down or inhibiting EZH2 can lead to upregulation of SOX9 in LNCaP cells (Fig. 4a, b). We found that knocking down SOX9 can inhibit the activation of NF-κB signaling (Fig. 4c), while overexpression of SOX9 had the opposite effect (Fig. 4d). Importantly, knockdown of SOX9 can also significantly inhibit the activation of the NF-κB signaling induced by EZH2 inhibitors (Fig. 4e) and sensitized LNCaP cells to EPZ6438 treatment (Fig. 4f). Thus, these results indicated that SOX9 contributed to EZH2 inhibition-induced feedback activation of NF-κB signaling pathway.

### SOX9 regulates the expression of TNFRSF11A

**Fig. 5:**
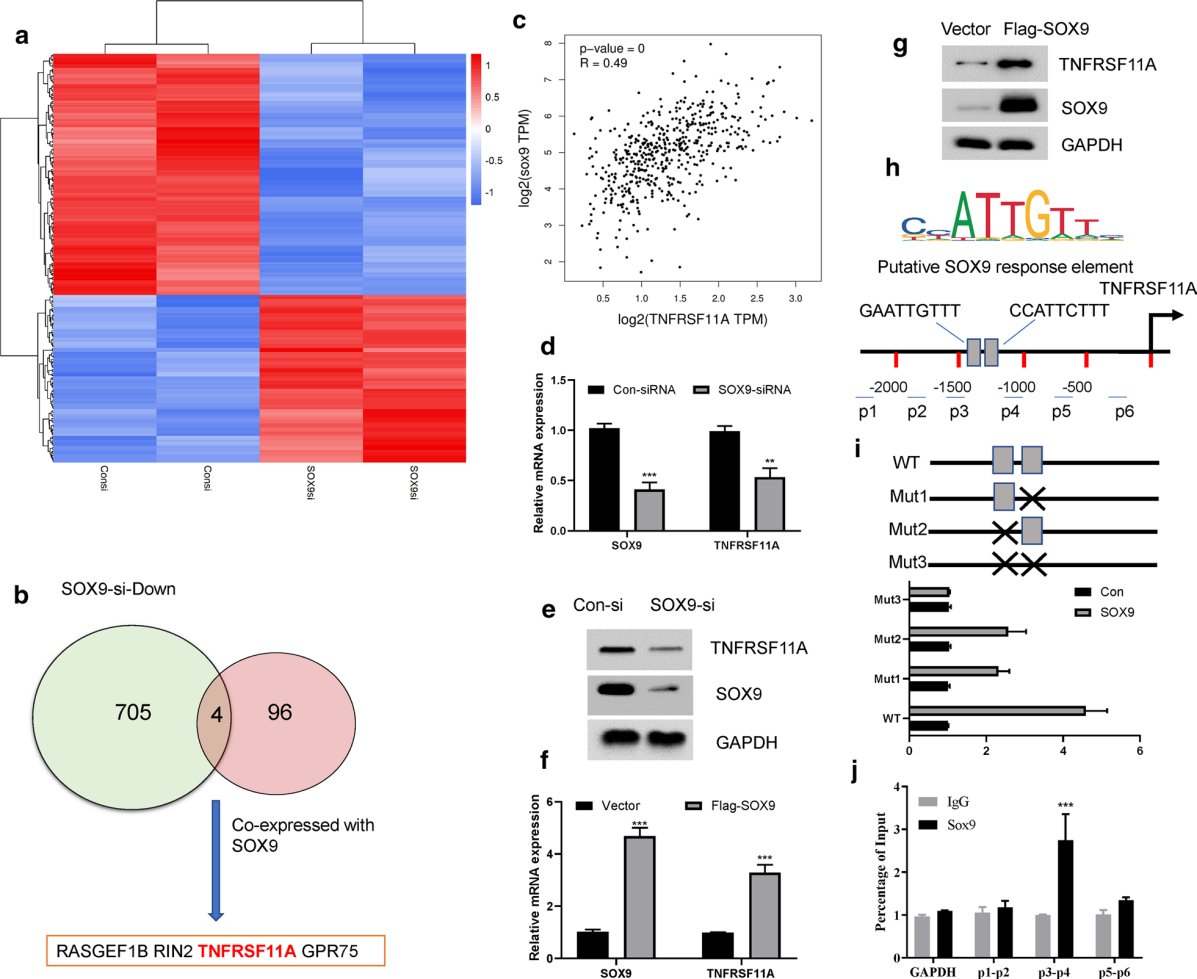
SOX9 regulates the expression of TNFRSF11A. **a** The microarray data from VCAP cells transfected with con-siRNA or SOX9-siRNA was used to examine the differentially expressed mRNAs. 1118 differentially expressed genes including 409 up-regulated and 709 down-regulated were identified by the Limma R package and the top 200 differentially expressed mRNAs were selected to draw a heatmap. **b** The intersection of the common genes between 709 down-regulated gene caused by SOX9 downregulation and 100 SOX9 co-expression genes identified 4 putative substrates of SOX9. **c** The correlation of the mRNAs of SOX9 and TNFRSF11A in 492 prostate cancer tissues from the TCGA database based on the GEPIA2 website (http://gepia2.cancer-pku.cn). **d** LNCaP cells were transfected with indicated siRNAs for 36 h, the relative mRNA expression levels of SOX9 and TNFRSF11A were determined by real-time PCR assay. **P < 0.01, ***P < 0.001. **e** LNCaP cells were transfected with indicated siRNAs for 36 h, and then subjected to western blot with indicated antibodies. **f** LNCaP cells were transfected with vector control or Flag-SOX9 plasmids for 36 h, the relative mRNA expression levels of SOX9 and TNFRSF11A were determined by real-time PCR assay. ***P < 0.001. **g** LNCaP cells were transfected with vector control or Flag-SOX9 plasmids for 36 h, and then subjected to western blot with indicated antibodies. **h** Schematic diagram shows the putative SOX9 response elements from the human TNFRSF11A gene promoter. **i** The human TNFRSF11A promoter contains two SOX9 response elements. Point mutation was highlighted with black cross. SOX9 was co-transfected with indicated plasmids into LNCaP cells for 36 h. The luciferase activity was then measured. **j** ChIP assay shows enrichment of SOX9 at the human TNFRSF11A gene promoter in LNCaP cells. The human GAPDH promoter was served as a negative control. ***P < 0.001. The primer pairs for ChIP assay were also demonstrated in **h**

Although NF-KB signaling could induce transcriptional up-regulation of SOX9 has been reported, how SOX9 regulates NF-KB signaling is still unclear. To this end, we first analyzed the GEO data of prostate cancer cells with or without SOX9 silencing (GSE76441) [19]. We chose a p < 0.05 and |log2FC| ≥ 1 as the cutoff criteria to select different expression genes. We found that 409 genes were up-regulated and 709 genes were down-regulated after SOX9 was silenced (Fig. 5a; Additional file 3: Table S3). We proposed that the direct transcriptional targets of SOX9 might be transcriptionally co-expressed in PCa tissues. Thus, we further selected the top 100 co-expressed genes of SOX9 from the TCGA prostate cancer database (Additional file 4: Table S4). The intersection of the two databases identified 4 putative substrates of SOX9, including RASGEF1B, RIN2, GPR75 and TNFRSF11A (Fig. 5b). TNFRSF11A, also known as receptor activator of NF-κB (RANK), is the receptor for RANKL. The RANKL-RANK signaling could significantly activate the NF-κB pathway and plays a critical in virous cancer types [20, 21]. Thus, we then focused on how TNFRSF11A was regulated by SOX9. As expected, the mRNA levels of SOX9 and TNFRSF11A were highly correlated in 492 PCa tissues from the TCGA database (Fig. 5c). Silencing the expression of SOX9 by siRNA in LNCaP cells significantly reduced the mRNA and protein expression of TNFRSF11A (Fig. 5d, e). On the contrary, overexpression of SOX9 by promoted TNFRSF11A expression at both mRNA and protein levels (Fig. 5f, g). Two Putative SOX9 response elements were found by analyzing the promoter region of TNFRSF11A gene (Fig. 5h). Through luciferase experiments, we found that overexpression of SOX9 can promote the activation of the wild-type (WT) TNFRSF11A promoter. Mutation of one putative SOX9 response element can reduce the luciferase activity, and mutation of both elements can completely block the luciferase activity (Fig. 5i). Moreover, chromatin immunoprecipitation (ChIP) assays showed abundant occupation of SOX9 at the human TNFRSF11A promoter where the putative SOX9 response elements located (Fig. 5j). Therefore, these data indicated that TNFRSF11A is a downstream target of SOX9 in PCa cells.

### Silencing of TNFRSF11A rendered PCa cells to EZH2 inhibitors treatment

**Fig. 6:**
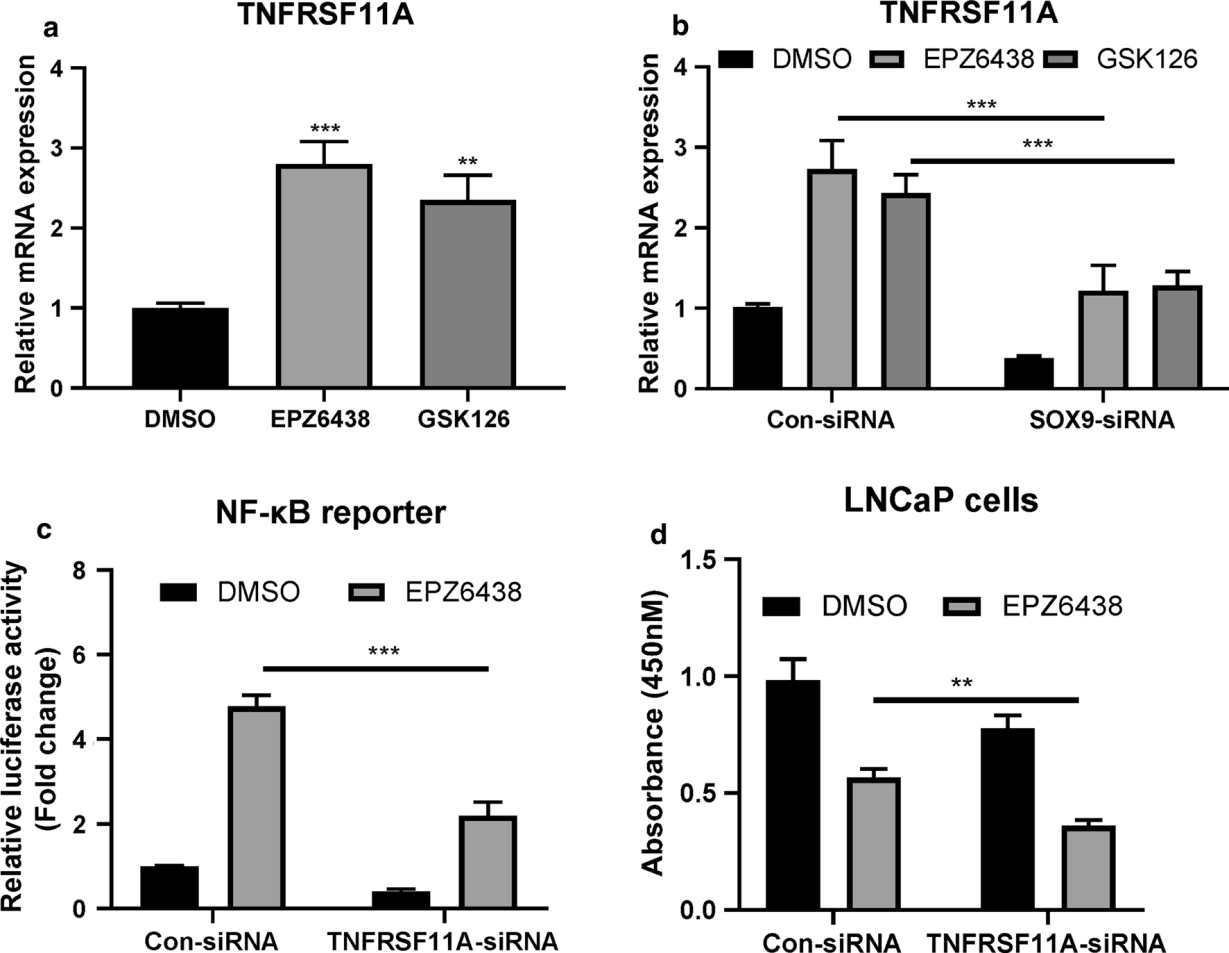
Silencing of TNFRSF11A rendered PCa cells to EZH2 inhibitors treatment.** a** LNCaP cells were treated with 10 μM EPZ6438 or GSK126 for 24 h. The relative mRNA levels of TNFRSF11A were then determined. **P < 0.01, ***P < 0.001. **b** LNCaP cells were transfected with indicated siRNAs for 36 h, the relative mRNA expression levels of TNFRSF11A were determined. ***P < 0.001. **c** LNCaP cells were transfected with indicated siRNAs for 12 h, then co-transfected with pGL3-NFkB-Luc and pSV40-renilla plasmids for 12 h and treated with or with EPZ6438 for additional 24 h. The luciferase activity was then measured. ***P < 0.001. **d** LNCaP cells were transfected with indicated siRNAs for 12 h and treated with or with EPZ6438 for additional 24 h. CCK-8 was then added and the absorbance at A450 was determined by a microplate reader. **P < 0.01

We next investigated whether EZH2 can regulate the expression of TNFRSF11A in PCa cells. We found that the mRNA levels of TNFRSF11A were significantly elevated in LNCaP cells followed EZH2 inhibitors treatment (Fig. 6a), which could be largely reversed when SOX9 was silenced (Fig. 6b). Moreover, we found that silencing of TNFRSF11A by siRNA in LNCaP cells prevented EPZ6438-induced NF-κB activation (Fig. 6c), and sensitized these cells to EPZ6438 treatment (Fig. 6d). Taken together, these results indicate that TNFRSF11A is involved in the resistance of PCa cells to EZH2 inhibitors treatment.

## Discussion

EZH2 can be used as the catalytic subunit of PRC2 to inhibit the expression of target genes in a H3K27me3-dependent manner, and it can also directly methylation several non-histone proteins, such as GATA4 (GATA binding protein 4) [22], AR (androgen receptor) and ER (estrogen receptor) [10]. Therefore, inhibiting the enzymatic activity of EZH2 alone may not sufficient to prevent the growth of tumor cells with high expression and/or mutation of EZH2. A recent study found that inhibiting the enzymatic activity of EZH2 can promote the transition from H3K27me to H3K27ac, which in turn leads to resistance of some tumor cells to EZH2 inhibitors [23]. However, H3K27ac co-inhibition improved efficacy of EZH2 inhibitors but also activates MAPK pathway in some cancers, suggesting that inhibition of EZH2 itself may feedback activation of some signaling pathways in a context-dependent manner [23]. Therefore, the discovery of EZH2-regulated signaling pathways that promote cancer cell survival will help set up novel tumor treatment strategies.

A previous study has reported that *S*-adenosylhomocysteine (SAH) can trigger the activation of NF-κB pathway to induce endothelial dysfunction and activation, by partially inhibiting the enzymatic activity of EZH2 [24]. We substantially extend earlier observations in PCa based on bioinformatics analysis from publicly available GEO database and revealed the molecular mechanism of how EZH2 regulates the NF-κB pathway. We show that inhibition of EZH2 in PCa cells induced the expression of the transcriptional factor SOX9, which in turn promoted the transcription of TNFRSF11A to promote the activation of NF-κB signaling. Our results reveal a EZH2-SOX9-TNFRSF11A axis in the regulation of NF-κB signaling in PCa cells, suggesting that targeting this axis could provide therapeutic promise. Indeed, interfering with this axis shows potent therapeutic potential for PCa treatment. For example, we showed that inhibition of NF-κB signaling by either BAY11-7082 administration or TNFRSF11A silencing could render PCa cells sensitive to EZH2 inhibitors.

SOX9 is a critical transcription factor in the SOX family, which plays an important role in embryonic development, maintenance of cell stemness and tissue homeostasis [25]. SOX9 has been identified as an oncogene, and its high expression is closely related to the occurrence of prostate cancer [26, 27]. In a recent report, SOX9 has been identified as a critical downstream target of EZH2 in rat cells. Inhibition of EZH2 decreased the level of H3K27me3 at SOX9 promoter region and increased SOX9 expression in rat endplate chondrocytes (EPCs) [17]. Consistent with this report, we show that EZH2 also regulates the expression of SOX9 in PCa. It has been reported that activated NF-κB signaling could induce the expression of SOX9. Thus, our results uncovered a positive feedback loop that increased SOX9 level also promote the activation of NF-κB signaling. Interestingly, our bioinformatics analysis found that after inhibiting EZH2, many viral infection-related processes were also activated, including COVID19, Epstein-Barr virus and viral protein interaction with cytokine and cytokine receptor (Fig. 1d). Although we do not yet know the reason for the specific activation of these processes, our findings may provide clues for further research on the biological function of EZH2 in the future.

## Conclusions

Together, our results reveal a critical link between EZH2 inhibition and NF-κB signaling activation in PCa, which might shed some lights on the clinical treatment of PCa patients.

## Supplementary Information

**Additional file 1.** The gene expression profile of LNCaP cells treated with siRNA against EZH2.

**Additional file 2.** The gene expression profile of LNCaP cells treated with a EZH2 inhibitor EPZ6438.

**Additional file 3.** List of 709 down-regulated genes in SOX9 silenced prostate cancer cells.

**Additional file 4.** List of top 100 co-expressed genes of SOX9 from the TCGA prostate cancer database.

**Additional file 5: Figure S1. **A PC3 cells were transfected with pGL3-NFkB-Luc and pSV40-renilla plasmids for 12 hr and then treated with indicated dose of GSK126 for additional 24 hr. The luciferase activity was then measured. ** P < 0.01, *** P < 0.001. B Relative mRNA expression levels of the two NF-κB downstream target genes c-Myc and Cyclin D1 in 10 μM GSK126 treated PC3 cells were determined by real-time PCR assay. *** P < 0.001. C Relative mRNA expression levels of the two NF-κB downstream inflammatory cytokines IL1B and TNFA in 10 μM GSK126 treated PC3 cells were determined by real-time PCR assay. *** P < 0.001.

## Data Availability

The microarray data (GSE107779 and GSE76441) were downloaded from the Gene Expression Omnibus (GEO) database.

## Abbreviations

CEP: Cartilaginous endplate
GEO: Gene expression omnibus
IL-1β: Interleukin-1β
PCa: Prostate cancer
PRC2: Poly-comb repressive complex 2
TNF-α: Tumor necrosis factor-α
