# Supplementary material for: Feedback activation of NF-KB signaling leads to adaptive resistance to EZH2 inhibitors in prostate cancer cells
Source: Cancer Cell Int. 2021 Apr 1;21:191. doi: 10.1186/s12935-021-01897-w (PMC8017762; doi:10.1186/s12935-021-01897-w)

Supplementary Figure 1

1. PC3 cells were transfected with pGL3-NFkB-Luc and pSV40-renilla plasmids for 12 hr and then treated with indicated dose of GSK126 for additional 24 hr. The luciferase activity was then measured. ** P < 0.01, *** P < 0.001.
2. Relative mRNA expression levels of the two NF-κB downstream target genes c-Myc and Cyclin D1 in 10 μM GSK126 treated PC3 cells were determined by real-time PCR assay. *** P < 0.001.
3. Relative mRNA expression levels of the two NF-κB downstream inflammatory cytokines IL1B and TNFA in 10 μM GSK126 treated PC3 cells were determined by real-time PCR assay. *** P < 0.001.


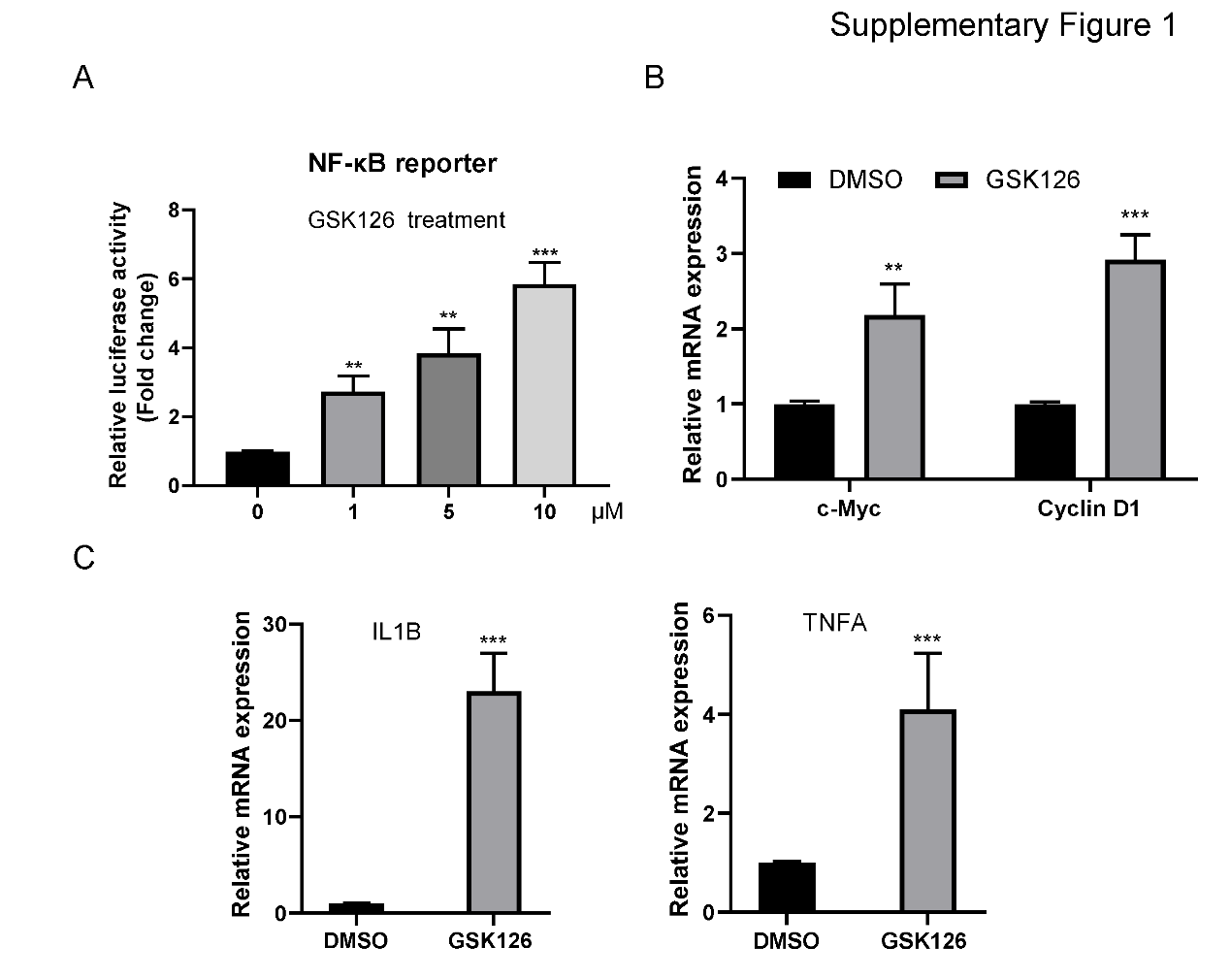

Supplement: Supplementary file 5 — Additional file 5: Figure S1. A PC3 cells were transfected with pGL3-NFkB-Luc and pSV40-renilla plasmids for 12 hr and then treated with indicated dose of GSK126 for additional 24 hr. The luciferase activity was then measured. ** P < 0.01, *** P < 0.001. B Relative mRNA expression levels of the two NF-κB downstream target genes c-Myc and Cyclin D1 in 10 μM GSK126 treated PC3 cells were determined by real-time PCR assay. *** P < 0.001. C Relative mRNA expression levels of the two NF-κB downstream inflammatory cytokines IL1B and TNFA in 10 μM GSK126 treated PC3 cells were determined by real-time PCR assay. *** P < 0.001. [file 12935_2021_1897_MOESM5_ESM.docx]
